# Pathogenic Mechanisms That May Link Periodontal Disease and Type 2 Diabetes Mellitus—The Role of Oxidative Stress

**DOI:** 10.3390/ijms25189806

**Published:** 2024-09-11

**Authors:** Jelena Mirnic, Milanko Djuric, Snezana Brkic, Ivana Gusic, Marija Stojilkovic, Ana Tadic, Tanja Veljovic

**Affiliations:** 1 Department of Dental Medicine, Faculty of Medicine, University of Novi Sad, 21000 Novi Sad, Serbia; marija.stojilkovic@mf.uns.ac.rs (M.S.); tanja.veljovic@mf.uns.ac.rs (T.V.); 2 Dentistry Clinic of Vojvodina, Department of Dental Medicine, Faculty of Medicine, University of Novi Sad, 21000 Novi Sad, Serbia; milanko.djuric@mf.uns.ac.rs (M.D.); ivana.gusic@mf.uns.ac.rs (I.G.); ana.tadic@mf.uns.ac.rs (A.T.); 3 Clinic for Infectious Diseases, Clinical Centre of Vojvodina, Department of Infectious Diseases, Faculty of Medicine, University of Novi Sad, 21000 Novi Sad, Serbia; snezana.brkic@mf.uns.ac.rs

**Keywords:** periodontal disease, diabetes mellitus type 2, oxidative stress, periodontopathogenic bacteria, mechanism, cytokine

## Abstract

Given the posited role of oxidative stress in the pathogenesis of both periodontitis and type 2 diabetes mellitus (T2DM), it may also serve as a link between these highly prevalent chronic inflammatory diseases. This view is supported by an ample body of evidence indicating that the severity and progression of periodontitis is in part driven by diabetes, while periodontal infection may hinder the attainment of adequate glycemic control in diabetic patients. Thus, this review focuses on the potential synergistic interactions along the oxidative stress–inflammation pathway characterizing both conditions. Because periodontitis and T2DM share the same risk factors and compromise patients’ quality of life, to develop effective strategies for combatting both conditions, their mutual influence needs to be explored.

## 1. Introduction

Both periodontitis and type 2 diabetes mellitus (T2DM) represent a significant global healthcare burden. Periodontal disease is estimated to affect up to 50% of the world population [1], and this figure is expected to increase in the future owing to the increased lifespan, greater retention of natural teeth, and rapid population aging [2]. The global diabetes epidemic is one of the biggest public health problems of the 21st century. Today, approximately 537 million people across the world suffer from diabetes, and the World Health Organization and the International Diabetes Federation estimate that the number of sufferers will increase to 643 million by 2030 [3].

Periodontitis is a condition caused by bacterial infection that leads to inflammation and destruction of the tooth-supporting tissues. Although more than 500 bacteria species can contribute to the development of dental plaque, *Porphyromonas gingivalis*, *Tannerella forsythia*, *Prevotella intermedia*, and *Aggregatibacter actinomycetemcomitans* are identified as the main causative agents of periodontitis. Still, the degree of destruction caused by these microorganisms is determined by their interaction with the host immune system [4], which is in turn influenced by genetic and environmental factors. According to the available evidence, diabetes is one of the key environmental risk factors for periodontitis [5].

Diabetes mellitus (DM) is an umbrella term referring to a range of metabolic disorders that manifest as an inability to control blood sugar as a result of defects in insulin secretion, action, or both [6]. Empirical evidence indicates that, over time, chronic hyperglycemia can lead to damage, dysfunction, and failure in various organ systems. The most common chronic complications in patients with diabetes are of a vascular nature and can be both macro- and microvascular in origin. Macrovascular complications, such as cardiovascular, cerebrovascular, and peripheral atherosclerosis [7], involve large blood vessels, and their main pathohistological substrate is atherosclerotic plaque. Cardiovascular complications are the leading cause of morbidity and mortality in patients with diabetes. It is estimated that about 32% of patients with diabetes suffer from some kind of cardiovascular disease, and about 50% of deaths in this population are attributed to these conditions [8]. Microvascular complications involve the smallest blood vessels, capillaries, and precapillary venules. Their main manifestation is microangiopathy—thickening of the capillary basal membrane due to the deposition of glycogen, mucopolysaccharides, and glycoproteins. Accordingly, they lead to conditions such as diabetic retinopathy, nephropathy, and neuropathy, resulting in various degrees of kidney failure and visual impairment [9].

Although many individuals living with T2DM suffer from periodontitis, the factors contributing to its development in this population are still not fully understood. Nonetheless, advances in periodontal medicine shed light on the role of inflammation and destructive processes in periodontal tissue in a number of systemic disorders. Accordingly, periodontal disease is increasingly being recognized as a risk factor for the emergence or exacerbation of some common diseases, including the inability to achieve adequate metabolic control in diabetes [10].

Guided by these findings, in this review, the potential synergistic interactions along the oxidative stress–inflammation axis common to both periodontitis and T2DM are explored.

## 2. Methods

To identify recent literature sources (published from 1 January 2000 to 1 April 2024) pertaining to the oxidative stress–inflammation axis, and specifically those exploring T2DM and periodontitis, we consulted Medline, Web of Science, and Scopus electronic databases. All searches were performed in Boolean/Phrase mode using the same MeSH terms and free text words, and were restricted to sources published in English. The following search terms were utilized: (“diabetes mellitus type 2” OR (MH “Diabetes Mellitus, Type 2”)) AND ((MH “Periodontal Diseases”) OR (MH “Periodontitis”)) AND (“periodontopathic bacteria” OR “periodontopathogens” OR “mechanism*” OR “oxidative stress” OR (MH “Oxidative Stress”) OR “cytokine*” OR (MH “Cytokines”)). This strategy yielded 622 original articles and reviews (453 from Scopus, 151 from Medline, and 18 from Web of Science). After title and abstract screening, 509 of these sources were eliminated, leaving 113 for a more detailed analysis. Only studies about the association between periodontal disease and diabetes mellitus type 2, or vice versa, were eligible for inclusion. Publications that met any of the following exclusion criteria were also eliminated: only abstract available, case report, letter to the editor, irrelevant study population (periodontal therapy in the previous six months, use of antibiotics in the last three months, use of any vitamin supplementation, pregnancy), inclusion of participants younger than 18 years, and studies considering both type 2 and type 1 diabetes mellitus. The quality assessment of included studies and data extraction were performed by two independent reviewers. Studies were evaluated and summarized in a narrative review.

## 3. Oxidative Stress

**Figure 1:**
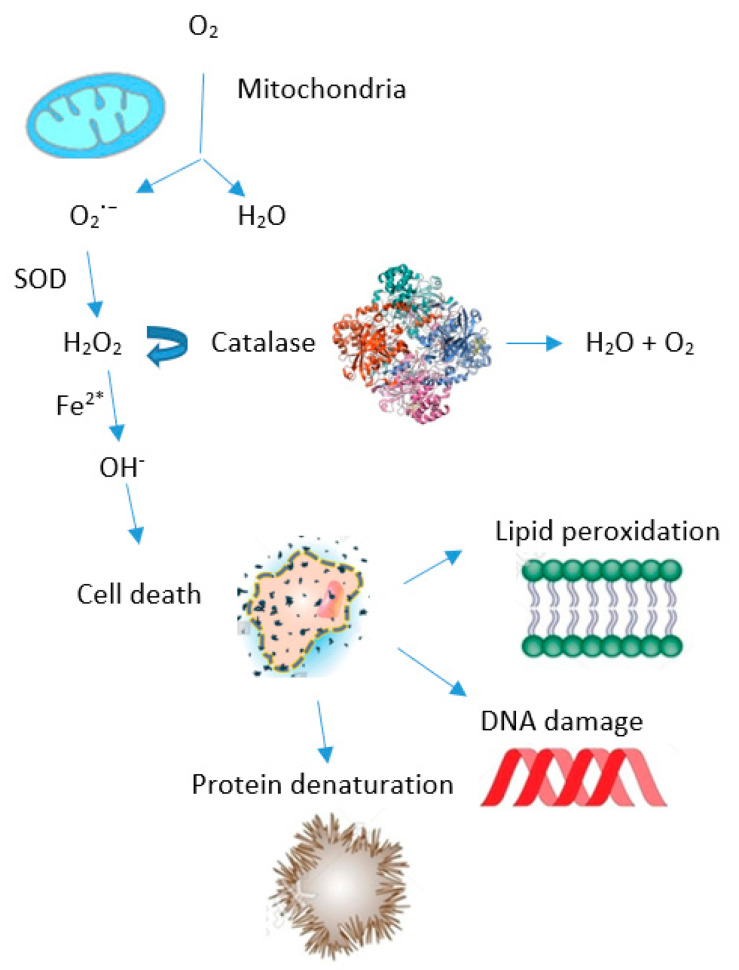
Generation of reactive oxygen species (ROS) and production of natural enzymes as an organism’s self-defense response. The ROS levels are governed by mitochondrial activity. The process commences with the conversion of superoxide radicals O2^·−^ to hydrogen peroxide—H_2_O_2_ (which is less toxic) by the superoxide dismutase (SOD) enzyme. Some of these H_2_O_2_ molecules—in the presence of ferrous ion Fe^2+^ are reduced to hydroxyl radical—OH^·^. This highly reactive ROS attacks various biomolecules (proteins, deoxynucleotide acid—DNA and lipids) and causes cell death. By blocking this pathway and decomposing H_2_O_2_ into harmless water and oxygen, catalase provides a defense to the organisms (modified from Rakotoarisoa et al. [13]).

In the human body, free radicals are constantly created, and they fulfill their physiological role of regulating cell signaling, activating receptors, protecting cells from various harmful effects, etc, at low concentrations. However, when the cellular redox homeostasis is disrupted due to excessive free radical formation and/or inadequate removal, oxidative stress occurs. This adverse effect arises because the “surplus” free radicals cause damage to cellular lipids, proteins, and nucleic acids (Figure 1). In addition, free radicals can change signal transduction and gene expression, thus contributing to pathological processes in the body [11]. It has also been established that free radicals participate in the pathogenesis of diabetes mellitus and HIV infection, as well as autoimmune, neurodegenerative, coronary, malignant, pulmonary, inflammatory, and many other diseases [12].

Free oxygen radicals represent the first line of defense against infectious agents. During the process of phagocytosis, an “oxidation explosion” occurs in activated polymorphonuclear leukocytes and macrophages due to up to 20-fold increased oxygen (O_2_) consumption and excessive superoxide anion radical (O_2_^·−^) and hydroxyl radical (OH˙) production, as well as increased creation of hydrogen peroxide (H_2_O_2_) and other reactive oxygen species (ROS), with the aim of destroying microorganisms. In the process of the “oxidation explosion”, more than 90% of oxygen in phagocytes is metabolized into O_2_^·−^ via the nicotinamide-adenine-dinucleotide-phosphate (NADPH) oxidase enzyme complex. The excess ROS that are released as a result do not selectively target the inflammatory response initiators, but also damage the surrounding healthy tissue (Table 1) [14].

In addition to phagocytes, many other cell types such as fibroblasts, lymphocytes, platelets, and endothelial cells contain NADPH oxidase and produce O_2_^·−^ in smaller amounts. Available evidence confirms that, in some of these cells, reactive oxygen metabolites produced in the NADPH oxidase system participate in signaling processes. Signal transduction is a process by which signals are transmitted from the cell surface into its interior [11]. Free radicals play an important role in the complex system of information transmission through the cell to the target molecules. In turn, via the initiation of gene expression and the subsequent synthesis of appropriate functional or structural proteins, these molecules enable the cell to adapt and survive, whereby free radicals serve as the cellular response initiators, transmitters, and/or modifiers [12].

Because ROS are very small, highly reactive, and diffusible molecules, they are ideal intracellular messengers. Specifically, transcription nuclear factor kappa B (NF-kB) is a regulatory protein that controls the expression of many inducible and tissue-specific genes. As such, it participates in the regulation of the cell’s immune response, as well as cell proliferation and apoptosis. Moreover, NF-kB activates genes that encode the synthesis of various cytokines (e.g., tumor necrosis factor-α (TNF-α), Interleukin (IL)-1, IL-2, IL-6), cytokine receptors, and cell adhesion molecules, including vascular cell adhesion molecule-1 (VCAM-1) and inducible nitric oxide synthase (iNOS). While NF-kB can be activated by numerous signaling molecules (including TNFα, IL-1β, viruses, and lipopolysaccharides), agents that cause oxidative stress are of particular relevance for the present discussion [15]. In this context, glucose and its oxidative products are noteworthy, as their activation of NF-κB is considered a key event in the transformation of the vasculature in diabetic patients [16]. In addition to NF-kB, the most important redox-sensitive proteins include the tyrosine kinase receptor class, especially the insulin receptor (β subunit) due to its tyrosine kinase activity [17].

## 4. Effect of Diabetes Mellitus on Periodontal Disease

### 4.1. The Role of Oxidative Stress

Although the relationship between diabetes and periodontitis at the cellular level is not fully understood, it is believed that oxidative stress plays a significant role in this link [12].

The DM onset and subsequent development of complications are closely related to the imbalance in the oxidative and antioxidant status of cells. Hyperglycemia is the main cause of the increase in the free radical concentration in the plasma of diabetic patients. The process of non-enzymatic glycolysis, in which the advanced glycation end products (AGEs) are formed, is accompanied by the creation of free oxygen radicals (O_2_^·−^, H_2_O_2_, OH˙). In addition, non-enzymatic glycolysis of extracellular and intracellular superoxide dismutase (SOD), as well as other anti-oxidative enzymes, reduces their catalytic activity. AGEs bind to receptors present on the surface of endothelial cells, macrophages, and neutrophils, which induces free radical generation and increased cytokine production by monocytes and endothelial cells [18]. A positive correlation has also been established between the degree of metabolic control attained by DM patients, the amount of released superoxide anion radical (O_2_^·−^) by neutrophils, and the extent of periodontal damage. These findings suggest that oxidative stress could be the mechanism responsible for the progression of periodontitis in individuals living with diabetes [19].

These assertions are supported by the results yielded by experiments involving animals. Namely, Ohnishi et al. [20] found significantly higher H_2_O_2_ values in the plasma of mice with experimentally induced diabetes compared to healthy controls, as well as an increase in alveolar bone resorption with the development of diabetes. Moreover, after the addition of an antioxidant (N-acetyl cysteine) to the food given to mice with diabetes, the authors recorded a decrease in plasma H_2_O_2_ levels along with the cessation of alveolar bone resorption.

### 4.2. Clinical and Epidemiological Studies

Available evidence indicates that the prevalence and the risk of more extensive damage to the periodontium are increased in diabetes [21,22]. For example, Nascimento et al. [21] found that diabetes increases the likelihood of the occurrence and progression of periodontitis by almost two-fold. In addition, according to the findings yielded by the meta-analysis of epidemiological studies conducted by Zheng et al. [22], periodontitis occurs in 68% of DM patients, nearly double the figure recorded for systemically healthy individuals (36%). Moreover, in the study conducted by Wu et al. [23], the periodontal status of T2DM patients was significantly worse compared to those without diabetes, as the former group had 0.61 mm deeper periodontal pockets, 0.89 mm greater attachment loss, and fewer natural teeth.

However, not all DM patients have the same risk of developing periodontitis. It is considered that the degree of metabolic control in these individuals is a very significant risk factor for periodontal damage, since hyperglycemia is the primary cause of the characteristic DM complications [24]. For example, by analyzing the data obtained via the National Health and Nutrition Examination Survey for the 2009–2014 period, Eke et al. [25] found that, in the U.S., people with poor glycemic control have a higher risk of developing periodontitis compared to systemically healthy controls. It is also believed that the degree of periodontal destruction largely depends on the degree of metabolic control of diabetes, since more extensive periodontal damage is usually diagnosed in DM patients that have failed to achieve adequate metabolic control, compared to those with good control [26,27]. For example, Lim et al. [28] found a significantly higher frequency of periodontal pockets ≥ 5 mm, and Aoyama et al. [29] reported worse mean CAL (clinical attachment level) and BOP (bleeding on probing) values for patients with poor metabolic control of diabetes compared to those with good control.

In addition to the degree of periodontium destruction, the response to periodontal therapy also depends on the degree of metabolic control of diabetes. Research shows that subjects with poorly controlled diabetes respond less well to periodontal therapy compared to subjects who do not suffer from diabetes, while periodontal therapy is equally effective in those with well-controlled diabetes and the controls [30,31].

However, these findings are countered by several studies where this correlation was not established [32,33,34], including the research conducted by Kardesler et al. [34], who reported similar probing depth (PD), CAL, and BOP values for subjects with periodontitis who had well-controlled and poorly controlled diabetes. These discrepancies are expected, as numerous risk factors play an important role in the degree of periodontal damage—primarily genetics, gender, age, socioeconomic status, and smoking—due to which the relationship between periodontal destruction and the metabolic control of diabetes is difficult to establish. In clinical practice, it is not uncommon to see patients with DM and poor glycoregulation without extensive damage to the periodontium, while others may have well-controlled diabetes but present with a high degree of periodontium destruction [35]. It is also worth noting that the results obtained in certain investigations do not support the view that the degree of metabolic control of diabetes affects the periodontal treatment success [36,37,38]. Indeed, several studies suggest that, in the short term, periodontal treatment is equally effective in T2DM patients and those without diabetes, but periodontal disease is more likely to reoccur in the former group when glycemic control is inadequate [30,39,40].

It is important to note that meaningful comparisons of the results yielded by different studies are challenging due to the use of different glycated haemoglobin (HbA1c) threshold values for the assessment of the degree of metabolic control by DM patients. Namely, although the strict limit values of this parameter were defined by the American Diabetes Academy in 2006 (HbA1c < 7% for well-controlled and HbA1c ≥ 7% for poorly controlled DM), these recommendations were not followed in all reviewed studies. As a result, subjects with the same HbA1c value (e.g., 8.0%) are considered as having good metabolic control in one study while being placed in the group with poor metabolic control of DM in other publications, which certainly affects the conclusions reached.

### 4.3. Pathogenetic Mechanisms of the Influence of Diabetes on Periodontal Disease

**Figure 2:**
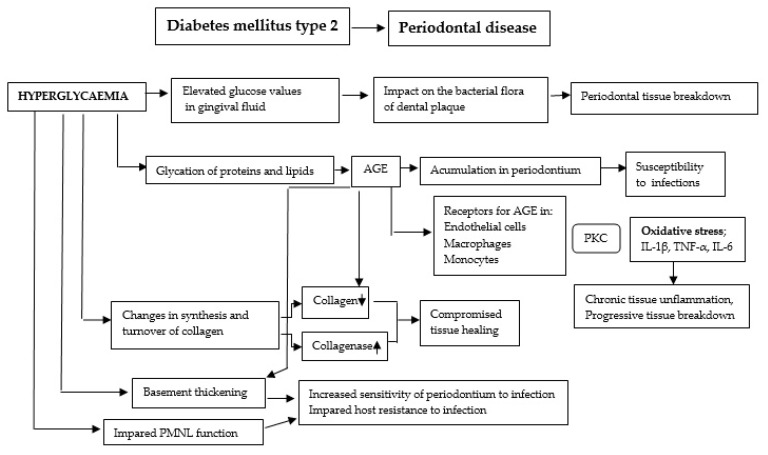
Potential mechanisms linking periodontitis exacerbation and hyperglycemia. Protein kinase C (PKC) is the predominant activation pathway for oxidative stress generation and proinflammatory cytokine production. AGEs—advanced glycation end-products; IL-1β—interleukin 1β; TNF-α—tumor necrosis factor-α; IL-6—interleukin 6; PMNL—polymorphonuclear leukocytes (modified from Kuo et al. [42]).

Although the pathogenetic mechanisms implicated in the development of periodontitis in individuals with diabetes have not yet been fully elucidated, vascular changes in the gingiva, immune mechanisms, collagen metabolism disorders, as well as specific microbiological flora in periodontal pockets are discussed in extant literature (Figure 2) [41].

#### 4.3.1. Vascular Mechanisms

The non-enzymatic glycosylation process by which glucose is chemically bound to the amino group of proteins without enzyme involvement is considered a very important mechanism for explaining DM complications, given that the degree of non-enzymatic glycosylation is directly related to the blood glucose level. Although HbA1c is the most studied glycosylated protein, other structural and regulatory proteins—such as serum albumin, collagen, and low-density lipoproteins (LDL)—undergo glycosylation by building advanced glycation end-products (AGEs). As the binding of glucose to proteins is highly stable, even if glycemic normalization occurs, it will not affect the already-formed AGEs. For this reason, AGEs accumulate in tissues, causing changes in cells and the extracellular matrix components [43]. In extant research, higher AGE accumulation was found in the periodontium of DM patients compared to healthy individuals [44]. Receptors for AGEs, so-called RAGE, are found on the surface of smooth muscle cells, endothelial cells, neurons, macrophages, and monocytes. As a result, RAGE expression is increased in hyperglycemia [45]. As the reactivity between AGE and RAGE on endothelial cells is also increased, so is the production of vascular endothelial growth factor (VEGF)—a multifunctional cytokine responsible for the regulation of vascular permeability and neovascularization. In turn, increased VEGF production increases blood vessel permeability. Thus, loss of the endothelial barrier represents an early pathophysiological mechanism in the occurrence of macro- and microangiopathy in DM [46,47]. In the capillaries of the periodontium, AGE-modified collagen accumulation results in the thickening of the basement membrane, which impairs the transport of oxygen, metabolic products, phagocytes, and antibodies, and thus contributes to the increased sensitivity of the periodontium in patients with diabetes [48].

#### 4.3.2. Immunological Mechanisms

Polymorphonuclear leukocytes (PMNL) represent the first line of defense against all forms of damage in the oral cavity, as well as in the entire organism. Due to the chemotactic movement towards the site of damage, phagocytosis, and digestion of microorganisms and their products, these cells play an important role in the body’s defense. It has been demonstrated that polymorphonuclear cell dysfunction has an unfavorable impact on the development of periodontitis [49]. In individuals with diabetes, impaired PMNL function has been found in chemotaxis, adherence, and phagocytosis, which leads to a weakened host response to infection [50,51].

As previously noted, hyperglycemia in T2DM patients increases oxidative stress and anti–inflammatory immune response, thereby exacerbating the periodontal disease. Specifically, in this population, the nuclear factor kappa B (NF-kB) is activated by the AGE–RAGE reaction, elevating oxidative stress and cytokine production [41,52]. Available evidence shows that oxidative deoxynucleotide acid (DNA) damage in the saliva of T2DM patients with periodontitis is more pronounced than in non-diabetic controls with periodontitis [53]. It is also noteworthy that in the study conducted by Arana et al. [54], T2DM patients with poor metabolic control had worse periodontal health as well as higher levels of salivary oxidative stress. In addition, lipid peroxidation markers (LPO) and cytokines (TNF-α, IL-6, and IL-10) in the gingival crevicular fluid were significantly elevated in T2DM patients with periodontitis compared to systemically healthy controls with periodontitis [55]. Moreover, Lalla et al. [56] induced a decrease in the level of cytokines (TNFα, IL-6) and alveolar bone resorption in diabetic mice after stimulation with *P. gingivalis* by blocking the RAGE. The dependence of the level of inflammatory cytokines in gingival tissue and gingival fluid on the degree of metabolic control of diabetes has also been determined [57]. For instance, Engebretson et al. [58] established that DM patients with HbA1c > 8% and periodontitis have almost twice higher IL-1β values in the gingival crevicular fluid (GCF) compared to patients with periodontitis and HbA1c < 8%. They concluded that hyperglycemia is independently associated with high GCF IL-1β levels (after adjusting for age, gender, and periodontal clinical parameters) in patients with type 2 diabetes and periodontitis.

Hyperglycemia also interferes with macrophage differentiation and secretion of growth factors (platelet-derived growth factor, basic fibroblast growth factor, transforming growth factor beta), which are necessary for tissue healing [59].

#### 4.3.3. Disorders in Collagen Metabolism

Collagen is the main constituent of the periodontal ligament and one of the key structural proteins in the periodontium. Because the periodontal ligament connects teeth to the alveolar bone, the progression of periodontitis is directly linked to its disorganization and destruction [49].

Both collagen synthesis and breakdown are thought to be influenced by glucose levels. For instance, during in vitro studies, the AGE–RAGE interaction has been shown to promote periodontal tissue destruction by inducing apoptosis of human periodontal ligament fibroblast cells and bone-forming osteoblasts [60,61]. Studies conducted on animal models similarly show that experimentally induced diabetes increases the activity of matrix metalloproteinases (MMP), especially collagenases (MMP-1, MMP-8, MMP-13) in the gingiva [62].

Furthermore, in hyperglycemia, collagen undergoes a process of non-enzymatic glycolysis and cross-linking occurs between collagen molecules, which significantly reduces its solubility [43]. These processes lead to the accumulation of AGE-modified collagen in the periodontium of diabetic patients, which can significantly compromise their tissue-healing capacity following periodontal therapy [35].

#### 4.3.4. Specific Microbiological Flora in Periodontal Pockets

It is considered that elevated glucose values in the gingival fluid in DM patients represent an additional source of nutrition for subgingival microorganisms, and thus favor the development of certain bacteria [63]. In addition, diabetes is a risk factor for the occurrence of xerostomia, which compromises the bactericidal function of saliva and can also cause an increased accumulation of dental plaque [64]. Consequently, Zhou et al. [65] and Shi et al. [66] argue that T2DM could have an impact on the bacterial flora of the subgingival plaque, owing to the differences in its composition between T2DM patients and systemically healthy controls irrespective of the status of their periodontium. These findings indicate that T2DM patients are at an increased risk of developing periodontal disease. Furthermore, Aemaimanan et al. [67] stated that DM patients with poor glycemic control and periodontitis have a higher number of red complex bacteria (*Porphyromonas gingivalis, Treponema denticola,* and *Tannerella forsythia*) in the subgingival biofilm compared to systemically healthy patients with periodontitis, which is positively correlated with the HbA1c level. However, the results yielded by clinical studies are inconsistent, as in some cases, no difference was found in the composition of bacterial flora between DM patients and systemically healthy controls with periodontitis [68,69,70]. Moreover, a comparison of the results obtained in different studies is difficult due to the inconsistencies in the sampling methods, microbiological techniques applied, clinical protocols, and participant selection criteria.

Given that periodontal diseases involve complex processes comprising microbe–microbe and microbe–host interactions, and may be accompanied by systemic diseases, determining periodontal pathogenicity based on taxa is challenging [71]. Nonetheless, animal studies involving mice not only indicate that diabetes induces changes in the composition of the oral bacterial flora, but also show how the transfer of modified microbiota in germ-free mice leads to more extensive periodontal inflammation and bone loss in comparison to the transference of microbes from mice with normal glycemia, thus providing new insights into pathogenic mechanisms of diabetes [72].

In recent studies, focus is increasingly given to the role of dysbiosis in T2DM-associated periodontitis, as well as to the potential benefits of probiotic therapy in regulating not only the gut microbiota, but also glucose metabolism, thereby boosting the immune system and lessening the oxidative stress [73].

## 5. Effect of Periodontal Disease on Diabetes Mellitus

### 5.1. Possible Increase in Oxidative Stress as a Consequence of Periodontitis in Patients with DM

The role of oxidative stress in periodontitis has been postulated by several authors, given that higher oxidative stress markers in either saliva or blood and/or decreased antioxidant status are consistently reported in patients with periodontitis relative to healthy controls [74,75]. Increased oxidative stress in the blood of patients with periodontal disease can have adverse consequences for their systemic health. For example, Tomofuji et al. [76] established that lipopolysaccharide-induced periodontitis in experimental mice caused an increase in serum hydrogen peroxide levels and oxidative DNA damage in the liver. Likewise, according to the results reported by Ekuni et al. [77], oxidative stress resulting from periodontitis could play a significant role in the progression of atherosclerosis. As a part of their investigation, these authors determined that periodontitis in experimental mice causes an increase in lipid peroxidation markers in periodontal tissues and serum, as well as lipid accumulation in the aorta. Thus, it can be posited that periodontitis could have a similar effect in patients with diabetes. Indeed, studies in which a part of this link was explored indicate that periodontitis has a negative impact on the already compromised oxidative status of patients with diabetes and is associated with beta cell dysfunction [78,79]. In particular, Allen et al. [79] found that patients with diabetes and periodontitis have higher levels of markers of oxidative protein damage in the blood, reduced ß-cell function, and higher HbA1c levels compared to patients with diabetes and healthy periodontium.

### 5.2. Clinical and Epidemiological Studies

Extant observational studies indicate that periodontal infection increases the risk of DM and compromises the metabolic control in those already suffering from diabetes [80,81]. For example, Demmer et al. [80] demonstrated that the progression of periodontal disease as well as advanced periodontitis serve as reliable predictors of HbA1c progression in diabetes-free individuals, indicating that chronic infections may play a role in diabetogenesis. Furthermore, following their literature review focusing on studies investigating the impact of periodontal disease on glycemic control in T2DM patients, Genco et al. [81] concluded that periodontitis leads to the worsening of glycemic control over time. Their investigations also show that the diabetic complications in T2DM patients with more advanced periodontitis tend to be more severe compared to those with mild or no periodontitis [81]. An ample body of evidence further points to the link between the severity of periodontitis in DM patients and nephropathy, retinopathy, various cardiovascular complications, neuropathic foot ulceration, and death due to cardio-renal disease [82].

Likewise, the risk of chronic complications associated with T2DM is linked to poor metabolic control, whereas even a 1% reduction in HbA1c levels leads to significant improvements [83]. However, as establishing good glycemic control seems to be a challenge for most T2DM patients with periodontitis [84], they might benefit from periodontal therapy with the view of improving their metabolic control [85].

Nonsurgical periodontal therapy provided to T2DM patients as a part of the periodontal disease management generally results in improved periodontal status. On the other hand, it is uncertain whether this contributes to improvements in glycemic control, as both positive [86,87,88,89,90,91] and insignificant [92,93,94] impacts on changes in HbA1c levels have been reported.

For example, Koromantzos et al. [86] found a significant decrease in the HbA1c levels among T2DM patients with advanced periodontitis who completed the initial therapy, and attributed these benefits to the significant improvement in the clinical periodontal parameters. Conversely, Engebretson et al. [92] did not find any improvement in glycemic control in patients with T2DM with moderate to advanced periodontitis following nonsurgical periodontal treatment. When interpreting the findings presented here, it is important to appreciate that a direct comparison of findings reported by different authors is rarely possible due to the variations in study protocols, including sample size, participant selection criteria for the treatment and control group (baseline HbA1c, periodontal disease severity), and antibiotics use, which will inevitably affect the obtained results [92].

Likewise, meta-analyses that have been conducted to date have yielded inconsistent findings, which can partly be attributed to the differences in the inclusion criteria and research protocols. For instance, while the meta-analysis conducted by Teewu et al. [95] focusing on highly heterogenous studies indicates that HbA1c levels may be reduced through periodontal therapy, these conclusions are countered by both positive [96,97] and inconclusive [98] findings yielded by more recent meta-analyses involving only randomized clinical trials.

### 5.3. Mechanisms by Which Periodontal Disease Might Affect DM

Periodontal disease occurs due to the reaction between the bacteria in the dental plaque and the host immune system, resulting in the immune–inflammatory response that manifests as infiltration of periodontal tissues by neutrophils, macrophages, and lymphocytes, and the generation of cytokines (IL-1β, TNF-α, and IL-6), prostaglandin E_2_, destructive enzymes (matrix metalloproteinases), and ROS in high concentrations [99,100].

As periodontitis is an inflammatory disease, it can also have a systemic effect. The subgingival biofilm represents a constant source of lipopolysaccharides, other products of Gram-negative bacteria, as well as the bacteria themselves, which may gain access to deeper periodontal tissues and systemic circulation. The epithelium of the periodontal pocket is the only barrier between the biofilm and the connective tissue. Thin, often ulcerated epithelium layers allow for the penetration of bacteria into the connective tissue and blood vessels. In patients with moderate and severe forms of the disease, the total surface of the periodontal pocket epithelium that is in direct contact with the subgingival biofilm ranges from 15 to 20 cm^2^—comparable to the size of the palm of an adult hand [101]. Research shows that patients with periodontitis have a significantly higher level of serum markers of oxidative stress as well as inflammation markers (such as C-reactive protein (CRP), IL-1, IL-6, and fibrinogen) compared to healthy individuals, and that periodontal therapy, in addition to reducing the clinical signs of periodontal inflammation, also leads to their reduction in serum [102,103,104,105,106,107,108].

**Figure 3:**
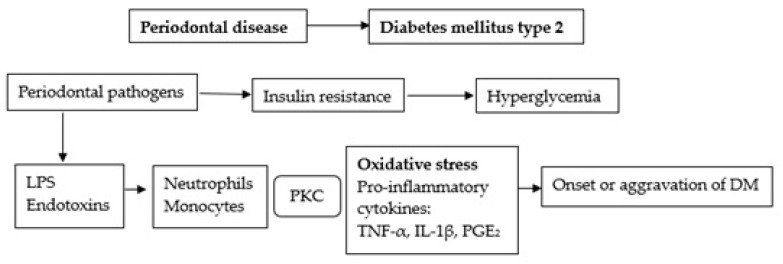
Potential mechanisms linking T2DM onset/exacerbation and periodontal disease. LPS—Lipopolysaccharide; Protein kinase C—PKC; TNF-α—tumor necrosis factor-α; IL-1β—interleukin 1β; PGE_2_—prostaglandin E_2_ (modified from Kuo et al. [42]).

Periodontal disease and obesity are posited to play similar roles in the increased insulin resistance in T2DM patients [35]. These arguments are based on the fact that TNF-α, IL-1, and IL-6 are produced in large quantities in adipose tissue and as a result of periodontal disease. The presence of pathogenic bacteria in periodontal tissues is believed to trigger cytokine production, as well as the release of reactive oxygen species that compromise insulin sensitivity or hinder its action over time (Figure 3) [85,109]. Hence, chronic inflammation resulting from periodontal infection could also contribute to insulin resistance and limit T2DM patients’ capacity to achieve adequate metabolic control, as well as lead to other related complications [81]. These assertions are supported by experimental studies involving animal models. For example, Watanabe et al. [110] demonstrated that periodontitis accelerates the onset of severe insulin resistance in Zucker Diabetic Fatty rats fed a high-fat diet. As the concentration of serum TNF-a was significantly higher in rats with periodontitis and T2DM compared to those with T2DM only, the authors posited that periodontitis and the associated production of proinflammatory cytokines might contribute to these findings. These researchers subsequently showed that lipopolysaccharides originating from periodontal pathogens bind to the Toll-like receptor 4, expressed by macrophages, hepatocytes, and pancreatic β-cells, which results in the upregulation in the transcription of proinflammatory cytokines such as TNF-α, IL-6, and IL-1β, thereby inducing insulin resistance [111].

These findings give credence to the view that, in patients with T2DM, glycemia may be exacerbated by periodontitis [79]. Accordingly, the reduction in circulating cytokine levels and oxidative stress achieved by periodontal treatment may lead to more optimal glycemic control [78,82,112,113]. However, data yielded by human studies are presently inconsistent, as the circulating levels of CRP, TNF-α, and IL-6 are found to be elevated in T2DM patients with periodontitis in some cases [114,115,116,117], but not in others [34,36,118]. Nonetheless, periodontal therapy is considered beneficial in reducing local oxidative stress and inflammatory marker levels in patients with periodontitis irrespective of their DM status [39,53,119]. Still, further research is needed to corroborate the limited evidence regarding its impact on the circulating reactive oxygen metabolites and inflammatory mediator levels in T2DM patients [39,113,120,121]. Based on the extensive literature review conducted as a part of this investigation, there is a paucity of comparative studies focusing on the oxidative status and periodontal therapy outcomes in systemically healthy individuals and those with underlying medical conditions such as DM. Nonetheless, as established by Gopalakrishnan et al. [113] and Marconcini et al. [120], reducing circulating oxidative stress through periodontal therapy increases the likelihood of more optimal glycemic control. It is noteworthy that, based on their systematic literature review and meta-analysis, Artese et al. [121] concluded that periodontal therapy reduces serum TNF-α and CRP levels in T2DM patients. Similarly, Nunez et al. [122] found that periodontal treatment improves metabolic control in T2DM patients, as well as the levels of specific cytokines. As other researchers failed to establish such a link, further research is required to resolve these discrepancies [123,124].

The strength of our study stems from the extensive research carried out using clear and appropriate inclusion and exclusion criteria when selecting the studies for review. Its main limitation is the small sample size, which was inevitable due to the small number of publications in which oxidative stress in patients with T2DM who also suffer from periodontitis is examined. This issue was further compounded by the high heterogeneity among the majority of the evaluated studies and the impossibility of standardizing the available data, which prevented us from conducting a meta-analysis.

## 6. Clinical Implications

According to the available evidence, the risk of developing periodontitis among T2DM patients is greater than in the general population and is related to the degree of metabolic control. Moreover, extant research indicates that untreated periodontitis can adversely impact metabolism, thereby increasing the likelihood of systemic health complications. In addition, periodontal therapy could contribute to HbA1c reduction [71].

Given these findings, it is essential to gain a greater understanding of the relationship between DM and periodontal disease and share these insights not only with healthcare professionals but also with DM patients. In particular, oral and periodontal healthcare must be recognized as an integral part of DM management, which should be conducted by the endocrinologist in collaboration with the dentist. Even if periodontal disease is not suspected, diabetologists should refer all their patients for a periodontal examination, and dental professionals should be aware of their glycemic status. If intraoral examination does not lead to the periodontitis diagnosis, T2DM patients should be monitored regularly for any changes in their oral health. Those in whom periodontitis is diagnosed should undergo periodontal therapy immediately. In either case, it is necessary to educate all T2DM patients on the importance of oral health as this will ensure their compliance with the treatment and follow-up protocols [125].

It is hoped that ongoing research and proactive dissemination of the obtained findings will facilitate the development of effective preventive strategies that can be routinely applied in clinical practice. The ultimate aim of this comprehensive approach is to limit the damage from increased oxidative stress in diabetes, as well as to identify the most beneficial forms of adjuvant antioxidative therapy for the treatment of diabetes complications.

## 7. Future Directions

Authors of earlier review papers on the impact of T2DM on periodontitis consistently recognize alterations in the host immunoinflammatory response and AGE-RAGE axis as important contributing factors to the tissue destruction and impaired tissue repair potential in periodontitis associated with DM. Accordingly, there is a prevalent view that the composition of the periodontal microflora is not significantly influenced by T2DM [35,85]. This perspective is countered by recent evidence obtained by applying new technologies in the examination of bacterial species. Indeed, more recent research points to the possible difference in the composition of the bacterial flora between systemically healthy patients with periodontal disease and those who also suffer from T2DM, especially if their metabolic control is poor [65,66,67].

As already mentioned, new insights into pathogenic mechanisms of diabetes gained through animal studies also merit further investigations. The aim is not only to elucidate the causal relationship between diabetes and periodontitis, but also to evaluate the applicability of the obtained findings to human subjects [72]. Another beneficial future research direction pertains to the treatment strategies that are effective in restoring oral eubiosis and preventing dysbiosis. This research domain is still in the initial stages, but extends to probiotics with high antioxidant capacity, with the potential for their adoption into the long-term periodontitis management protocol for T2DM patients [73].

Given the paucity of studies focusing on the impact of periodontitis on diabetes, and especially those in which the effects of inflammation of the periodontium and periodontal therapy are explored, there is an evident need for large multicenter randomized clinical trials in which a full range of biomarkers should be examined. Emphasis should be placed on markers of oxidative stress to better understand the role of periodontal treatment in systemic inflammation and its link to hyperglycemia. Additional animal studies exploring the biological effect of periodontitis on DM models are also necessary.

## 8. Conclusions

Diabetes is recognized as one of the key contributors to the severity and progression of periodontitis, and its severity is linked to poor glycemic control. Because this relationship is bidirectional, in that advanced periodontitis may undermine adequate glycemic control, it is evident that DM patients need to be made aware of their increased risk of periodontitis and its potential negative impact on metabolic control, which would in turn increase their risk of diabetes complications. Thus, it is necessary to incorporate periodontal healthcare into DM management protocols, which requires multidisciplinary treatment of T2DM patients with periodontal disease. While treating periodontal infection in individuals with diabetes is clearly essential for maintaining oral health, it may also play an important role in establishing and maintaining glycemic control, potentially delaying or even preventing the onset or progression of diabetes complications. A better understanding of the pathogenetic mechanisms that link diabetes and periodontitis should contribute to the development of new therapeutic approaches that will reduce the risk of complications associated with both conditions.

## Figures and Tables

**Table 1:** Commonly used markers of oxidative damage.

	Markers of Oxidative Stress
Lipid peroxidation	Malondialdehyde (MDA) Thiobarbituric acid reactive substances (TBARS)
DNA (deoxynucleotide acid) oxidation	8-hydroxy-deoxyguanosine (8-OHdG)
Protein oxidation	Protein carbonyl
